# Covalently Anchored Molecular Catalyst onto a Graphitic Carbon Nitride Surface for Photocatalytic Epoxidation of Olefins

**DOI:** 10.1021/acscatal.4c04187

**Published:** 2024-09-18

**Authors:** Sebastiano Gadolini, Rachel N. Kerber, Riho T. Seljamäe-Green, Wenming Tong, Pau Farràs, Elena C. Corbos

**Affiliations:** † Johnson Matthey Technology Centre, Blounts Court, Sonning Common, Reading RG4 9NH, U.K.; ‡ School of Biological and Chemical Sciences, Energy Research Centre, Ryan Institute, University of Galway, University Road, Galway H91 CF50, Ireland

**Keywords:** photoepoxidation, heterogenous photocatalysis, catalyst recycling, iron salen complex, hybrid catalysis, alkenes oxidation, surface modification

## Abstract

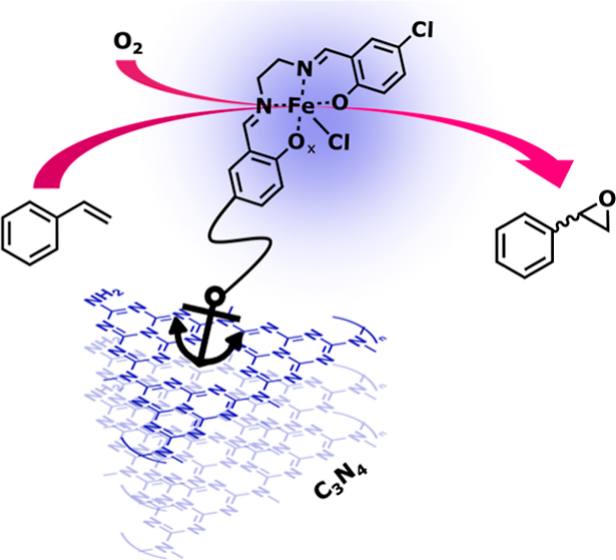

This study explores an innovative photocatalytic approach using pristine graphitic carbon nitride (C_3_N_4_) to anchor iron salen-type complexes (FeSalenCl_2_) without the need for additional linkers or heterojunctions. The resulting hybrid catalyst, [C_3_N_4_-FeCl(Salen)]_Chem_, exhibits a promising catalytic performance in the selective epoxidation of cyclic and linear olefins using gaseous oxygen as the oxidant. The catalyst’s selectivity closely resembles that of the free iron complex, and its effectiveness varies depending on the olefin substrate. Additionally, solvent selection plays a critical role in achieving optimal performance, with acetonitrile proving to be the best choice. The study demonstrates the potential of C_3_N_4_ as an environmentally friendly, recyclable, and efficient support for molecular catalysts. The results highlight the versatility and significance of C_3_N_4_-based materials in advancing light-driven catalysis.

## 1 Introduction

**Scheme 1:**
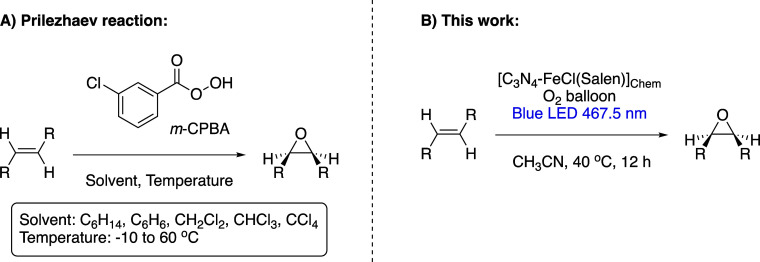
Epoxidation of Olefins via Conventional Prilezhaev Reaction and the Proposed Photocatalytic Alternative; (A) Prilezhaev Reaction, Epoxidation of Olefins: It Involves the Reaction of an Olefin with *meta*-Chloroperoxybenzoic Acid (*m*-CPBA) as an Oxygen Source, Inert Solvents (C_6_H_14_, C_6_H_6_, CH_2_Cl_2_, CHCl_3_, and CCl_4_), and a Range of Temperatures between −10 and 60 °C with the Yield of 60–80%; (B) This Work, Photocatalytic Epoxidation of Olefins, Involves Using an Iron Salen-Type Complex Anchored on the Surface of Graphitic Carbon Nitride as a Photocatalyst for Alkene Epoxidation; the Reaction is Performed in Acetonitrile at Room Temperature (the Light Source Warms up the Reaction to 40 °C) and in the Presence of Molecular Oxygen

Epoxides play a pivotal role as intermediate compounds in creating various crucial commercial products, attracting significant attention from the academic and industrial domains. Their importance lies in their capacity for ring–opening reactions, which are fundamental in crafting intricate organic molecules.^1−4^ Frequently, epoxides formed initially engage in further reactions to yield industrially valuable products, including surfactants, detergents, antistatic and anticorrosion agents, additives for laundry detergents, lubricating oils, textiles, and cosmetics. Epoxides are synthesized by adding oxygen to alkenes, employing electrophilic agents such as molecular oxygen, peroxy acids, hydrogen peroxide, or hydroperoxides in their free or chemically bound form.^1,5,6^ While molecular oxygen can be used for ethylene epoxidation, the majority of alkene epoxidation processes rely on stoichiometric quantities of alkyl hydroperoxides or peracids; this process is known as the Prilezhaev reaction,^7^ which is shown in Scheme 1A. The main drawback of using peroxy acids is that they lead to the undesired formation of alcohols or carboxylic acids as byproducts. Furthermore, peracids pose safety concerns due to their hazardous nature. Therefore, there is a pressing need to develop novel epoxidation techniques that utilize safer and more selective oxidants, such as hydrogen peroxide, molecular oxygen or water, in combination with the right catalyst and minimize the generation of waste products.^8−10^

In the pursuit of environmentally friendly epoxidation processes, photocatalysis emerges as a sustainable and safer alternative to conventional routes. By harnessing the power of light and semiconductor materials, photocatalytic systems can generate highly reactive species that facilitate the selective formation of epoxides from olefins.^11,12^ This innovative method aligns with the principles of green chemistry and has the potential to revolutionize epoxide production, addressing both safety and environmental concerns.^13^ Notably, the pioneering work of Inoue et al. marked a turning point, employing tetraphenylporphyrinantimony(V) as a sensitizer in the photochemical epoxidation of alkenes^14^ and sparking the development of numerous photocatalytic epoxidation systems. Similarly, developing manganese(V) nitrido complexes as efficient catalysts for visible-light-induced oxidation of alkenes and alcohols has opened avenues for constructing highly efficient photochemical oxidation catalysts.^15^ While homogeneous photocatalysts exhibit high activity and selectivity for oxidation reactions, their preparation and separation from reaction products and medium often entail significant energy and cost. Additionally, these materials tend to deactivate easily in oxidizing conditions due to the formation of oxo dimers and other polymeric species.^16^ To overcome these limitations, designing heterogenized metal complex catalysts onto solid support offers a cost-effective and environmentally friendly practical solution.^17,18^

The choice of the molecular catalyst is crucial to enhancing the practicality of such hybrid catalytic systems. Schiff base complexes, in particular, offer an attractive option due to their cost-effective preparation methods and versatility. In general, Schiff bases and their metal complexes are multipurpose compounds produced from the condensation of amines with carbonyl compounds and are extensively used in many research and industrial applications in analytical chemistry,^19^ dye industry,^20^ and corrosion inhibitors.^21^ Among them, salen-type complexes stand out for their affordability, ease of synthesis, and the ease with which they can be modulated, both in terms of steric and electronic properties.^22,23^ Researchers have developed various methods to create hybrid molecular-heterogeneous catalysts by modifying the ligand structure or the Supporting Information. Among these approaches, Schiff base complexes of manganese have garnered significant attention and are some of the most extensively studied molecular complexes for hybrid materials. They have been employed in diverse forms, including covalent and coordinated covalent binding to insoluble polymers,^24^ carbon nanotubes,^25^ porous materials,^26^ and silica.^27^ In the context of light-driven epoxidation, an intriguing development involves the successful immobilization of cobalt-solden-type complexes onto TiO_2_ nanoparticles. This innovative approach showcases their synergistic effect under UV-light irradiation.^28^ However, given the limitations of TiO_2_ as a heterogeneous photosensitizer, such as its redox potentials and the singlet–triplet energy gap, achieving high selectivity and reactivity under mild conditions can be challenging. The choice of the semiconductor drastically affects the performance of the target reaction.^29^ In fact, the energy required to transition from the lowest-energy singlet to the triplet states is a key property of photoactive materials because it influences their optical and electronic features.^30,31^ For instance, in photosystem II, chlorophyll absorbs light and can generate triplet states, which then interact with triplet oxygen to produce highly reactive singlet oxygen.^32^ Recent studies have shown that carbon nitride materials, especially in their graphitic form, exhibit an interesting phenomenon where the singlet exciton lies below the triplet state, contrary to what is commonly expected. This singlet–triplet energy inversion was observed by Actis et al. through time-resolved electron paramagnetic resonance spectroscopy, highlighting the unique photophysical properties of carbon nitride.^33^ This inversion suggests that singlet and triplet excitons are nearly isoenergetic, which could promote efficient singlet–triplet conversion, thereby enhancing photocatalytic performance under visible light irradiation.^33^

In this context, graphitic carbon nitride (C_3_N_4_) polymers have emerged as a promising alternative. These polymers have garnered significant attention, thanks to their remarkable attributes, including their ability to absorb visible light, high quantum yield, stability, low toxicity, and biocompatibility.^34,35^ C_3_N_4_ is a low-cost photocatalyst comprising carbon, nitrogen, and hydrogen within a poly(tri-*s*-triazine) framework with amino terminal groups.^36^ Notably, C_3_N_4_ can be readily doped or chemically functionalized, allowing for the fine-tuning of its photophysical properties.^37^ Common strategies involve the incorporation of transition metals via covalent or noncovalent interactions, which have shown remarkable results in various photocatalytic applications.^38−42^ These modifications of carbon nitride’s band gap and electronic structure enable a broader range of solar spectrum utilization, making it suitable for aerobic oxidation of organic substrates.^43−46^ Ding et al. showed that transition metal ions including Fe, Co, Ni, Mn, and Cu could be successfully included into a C_3_N_4_ matrix by a simple soft-chemical method; among the studied doped materials, Co- and Fe-modified carbon nitrides were proven to be active for the epoxidation of styrene with O_2_.^47,48^ Simaioforidou et al. have developed a strategy to alter the amino-terminal groups of C_3_N_4_ to be able to covalently support the manganese Schiff base complex for photocatalytic epoxidation of olefins.^49^

Building upon the promising attributes of graphitic carbon nitride as an alternative photosensitizer, our ongoing research explores an innovative approach that leverages the intrinsic capabilities of pristine C_3_N_4_ itself. In this work, we use the amino-terminal groups naturally present within C_3_N_4_ heptazine ring units as anchoring points for iron salen-type complexes, eliminating the need for additional linkers or the formation of heterojunctions with other semiconductors or Supporting Information. In our system, C_3_N_4_ is implemented as a light harvester and the transition metal complex is implemented as a catalyst. The role separation approach can simplify and enhance the photocatalytic potential of molecular-heterogeneous photocatalysts based on C_3_N_4_. We investigated the structural and optical properties of this new material by using Fourier transform infrared (FTIR), UV–vis, and microscopy techniques. Furthermore, we explored the immobilization and recycling effect of the molecular catalyst with semiconducting C_3_N_4_, beginning with the photocatalytic epoxidation of styrene and extending our study to cyclic and linear olefins with diverse electronic and structural properties. The reaction conditions are detailed in Scheme 1B. Our results show that chemical linkage between the iron salen-type complex and C_3_N_4_ provides high selectivity and activity toward the relevant epoxide using oxygen gas as the O source. Most importantly, the activity is retained after three catalytic tests recycling the photocatalyst via a simple centrifugation. It is important to highlight that no additives were added in our tests and all the activity arises from the photocatalyst alone, whereas other examples in the literature use isobutyraldehyde, without which no conversion is observed.^50^

## 2 Materials and Methods

### 2.1 Materials Availability

Commercially available reagents were purchased from Fluorochem and Fisher Scientific and used without further purification. All of the steps in the catalyst preparation were carried out in a dry nitrogen atmosphere using standard Schlenk techniques.

### 2.2 DFT Methodology

Density functional theory (DFT) was used to study the geometry, electronic structure, and spectroscopic properties of Melem (including its isomers), FeCl(Salen), and [Melem-FeCl(Salen)]_Chem_ materials. Turbomole (http://www.turbomole.com) was used to perform optimization using the def2-TZVP basis set and the HSE06 functional.^51−54^

The formation energy of [Melem-FeCl(Salen)]_Chem_ was calculated using the general reaction energy formula: E_r_ = {E([Melem-FeCl(Salen)]_Chem_) + E(HCl)} – {E(Melem) + E(FeCl(Salen))}.

For the UV–vis spectra calculations, the time-dependent DFT (TDDFT) method was employed to calculate the excitation energies and oscillator strengths of the system related to the absorption spectrum.^55^

### 2.3 Iron Salen-Type Complex FeCl(Salen) Preparation Procedure

The Schiff base ligand **SalenCl**_**2**_ was synthesized by the well-known reaction between salicylaldehyde and diamine, according to a published procedure.^56^ One equivalent of ethylenediamine (0.325 M) was added to a solution of two equiv of 4-chlorosalicylaldehyde (0.65 M) in ethanol at room temperature, and the mixture was stirred for 6 h to afford a yellow suspension. After filtration, the product was recrystallized from ethanol/CHCl_3_ to give a yellow, crystalline solid. SalenCl_2_, [1,2-bis(5-Cl-salicylideneamino)ethane]: yield 72%. ^1^H NMR (300 MHz, DMSO-*d*_6_): 11.11 (s, 2H, OH), 8.54 (s, 2H, HC = N), 7.60 (s, 2H, Ar–CH), 7.25 (m, 2H, Ar–CH), 6.96 (m, 2H, Ar–CH), 3.95 (s, 4H, CH_2_); IR [attenuated total reflection (ATR), cm^–1^]: v = 3081, 2943, 2905, 2851, 1631, 1479, 1361, 1273, 1034, 822, 776, 706, 644, 563. UV–vis (CH_3_CN, 0.1 mM, 25 °C): 312 and 360 nm. The corresponding iron complex **FeCl(Salen)** was prepared from Schiff base, anhydrous ferric chloride, and trimethylamine of 1:1:2 molar ratios in methanol and recrystallized from methanol/CH_2_Cl_2_.^56^ Yield 58%. IR (ATR, cm^–1^): 2988, 2971, 2923, 2902, 1628, 1471, 1445, 1243, 1046, 903, 754, 616, 584 (Fe–N), 562. UV–vis (CH_3_CN, 0.1 mM, 25 °C): 309 nm, 365 nm, 539 nm.

### 2.4 Hybrid C_3_N_4_-Salen Complex [C_3_N_4_-FeCl(Salen)]_Chem_ Catalyst Preparation

**C**_**3**_**N**_**4**_ was prepared via the thermal condensation of melamine. In detail, melamine was placed in a closed alumina crucible; then, it was heated to 550 °C in a muffle furnace with a heating rate of 2 °C/min and held at this temperature for 4 h in the air. After cooling to room temperature, the bulk C_3_N_4_ was crushed into a powder and refired at 500 °C for 8 h to exfoliate the material thermally. The **hybrid C**_**3**_**N**_**4**_**-salen complex [C**_**3**_**N**_**4**_-**FeCl(Salen)]**_**Chem**_ was prepared using a modified literature procedure.^57^ In a dry nitrogen atmosphere, a Schlenk tube was charged with C_3_N_4_ (200 mg), dry toluene (10 mL), and potassium *tert*-butoxide (12 mg). The iron complex (8 mg) was then added to the slurry mixture. The tube was then sonicated for 1 h, followed by reflux for 40 h. The tube was allowed to cool down, and 10 mL of water was added, followed by 30 mL of DMSO. The solid was then separated by centrifugation (5000 rpm, 30 min). The final product was washed with water to remove the remaining absorbed catalyst off the C_3_N_4_ surface. The resulting catalyst was dried overnight at 80 °C. C_3_N_4_-(SalenCl_2_) was synthesized by the same procedure with the free ligand instead of FeCl(Salen).

### 2.5 C_3_N_4_ with Physically Absorbed Salen [C_3_N_4_-FeCl(Salen)]_Phys_ Complex Preparation

**FeCl(Salen)** (8 mg) was dissolved in CH_2_Cl_2_ (5 mL), and separately, **C**_**3**_**N**_**4**_ (200 mg) was suspended in ethanol (5 mL). The complex solution was added dropwise to the C_3_N_4_ suspension under stirring. The solvent was removed by rotary evaporation, and the solid was dried under reduced pressure overnight without further purification.

### 2.6 Sample Characterization

UV–vis spectrometry measurements were carried out on an Agilent Cary 5000 spectrophotometer (200–2500 nm range) with a deuterium UV lamp light source using R928PTM. Reflectance measurements were recorded with a solid sample cell holder. Liquid measurements were recorded in 1 cm wide quartz cuvettes and performed at different temperatures (depending on the type of measurement) using a Peltier Dual Cell Holder. IR spectra (4000–650 cm^–1^) were recorded using a PerkinElmer 16PC FT-IR spectrometer with a KBr reference and a Bruker Alpha ATR-FTIR. AQF-IC data were collected using a Mitsubishi AQF-2100H. Inductively coupled plasma mass spectrometry (ICP-MS) data were collected using an Agilent 7700 ICP-MS. Scanning electron microscopy-energy-dispersive X-ray spectroscopy (SEM-EDX) measurements were carried out on a Hitachi S-4700 SEM instrument with a Bruker XFlash 6160 EDX detector. X-ray photoelectron spectroscopy (XPS) data were collected with a Thermo NEXSA. ^1^H NMR spectra were recorded at 300 MHz using a Bruker spectrometer and were processed using Bruker Topspin software with calibration against solvent peaks, according to published values.

### 2.7 Photocatalytic Epoxidation of the Olefins Procedure

The photocatalytic reactions were carried out in a SynLED parallel photoreactor (https://www.sigmaaldrich.com/deepweb/assets/sigmaaldrich/product/documents/398/704/z742680bul.pdf) with Wheaton scintillation vials (8 mL). The equipped blue light-emitting diode (LED) (12 W) with a 465–470 nm wavelength was employed as the light source. Typical conditions for epoxidations were as follows: 5 mg of catalyst, or 0.08 mg/mL of complex, was loaded into the reaction vessel with a magnetic stirrer. The vial was then purged with nitrogen for 30 min and sealed with a rubber septum. After addition of 5 mL of acetonitrile and 1.0 mmol of olefin, the mixture was irradiated and exposed to different oxidizing agents for 12 h at 40 °C (temperature reached by the photoreactor after the reaction time). The olefin substrates used in this study included cyclic and linear olefins, such as styrene, cyclohexene, α-pinene, 1-octene, and *cis*-4-octene. In the case of gaseous oxygen, the vial was equipped with an oxygen balloon. In the case of hydrogen peroxide, 3 equiv of H_2_O_2_ 30% was added to the mixture. Control experiments were performed without a catalyst, in homogeneous conditions, in dark and inert atmospheres. After the reaction, the stability of the catalyst was investigated by centrifuging the suspension, washing it with acetonitrile, and drying the vial at 80 °C overnight. Products of epoxidation were analyzed with a gas chromatograph coupled with mass analysis. Both the conversion and selectivity were obtained through standard calibration curves. The formula is listed below.
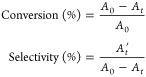
where *A*_0_ is the peak area of the olefin determined by GC before reaction, *A*_*t*_ is the peak area of the olefin after the reaction for *t* hours, and *A*′_*t*_ is the peak area of the epoxide after the reaction for *t* hours.

### 2.8 In Situ Monitoring of the Complex Leaching Procedure

The reaction conditions matched those described earlier for hydrogen peroxide as the oxidizing agent. The experiments were conducted by using an Agilent Cary 5000 spectrophotometer equipped with a Peltier Dual Cell Holder. The cell holder facilitated controlled temperature and stirring measurements for the sample and a reference. In this setup, two cells were loaded with CH_3_CN (2.5 mL), H_2_O_2_, and styrene, with the hybrid catalyst (5 mg, of which 0.2 mg is the theoretical maximum of complex loading or 0.08 mg/mL in solution) introduced into the sample cell. Additionally, C_3_N_4_ (5 mg) was used as the reference to subtract its contribution from the background. The experiment was carried out at 40 °C for a duration of 4 h, with spectra recorded at 20 min intervals until an overlap of signals was observed, indicating that a plateau was reached for the concentration of the complex that leached in solution. From the results (Figure S8), 0.03093 mg/mL of the complex was found in the solution. The approximate amount of the complex that was leached out is 0.077 mg, 38.7%.

## 3 Results

### 3.1 Material Synthesis and Characterization

**Scheme 2:**
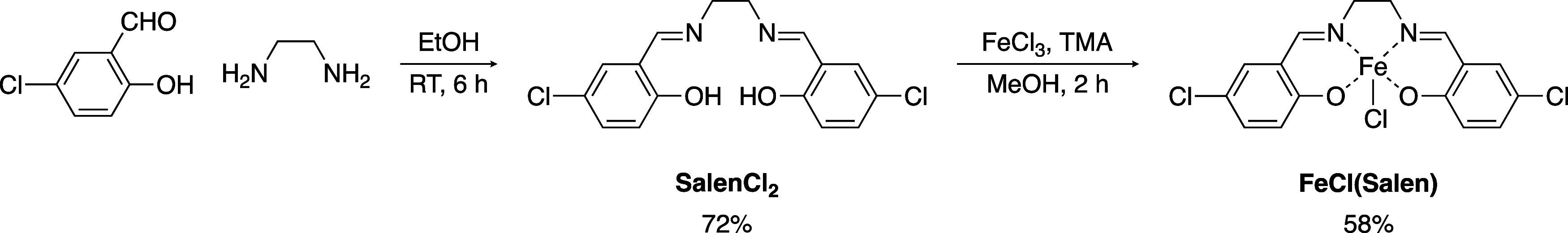
FeCl(Salen) Complex Synthetic Strategy; the Schiff Base Ligand SalenCl_2_ Was Synthesized by Condensation between 4-Chlorosalicylaldehyde and Ethylenediamine in Ethanol; the Corresponding Iron Complex Was Prepared from the Schiff Base, Anhydrous Ferric Chloride, and Trimethylamine (TMA) with 1:1:2 Molar Ratios in Methanol

The Schiff base ligand SalenCl_2_ was synthesized via the condensation of 4-chlorosalicylaldehyde and ethylenediamine in ethanol. The corresponding iron complex was prepared using the Schiff base, anhydrous ferric chloride, and trimethylamine with 1:1:2 molar ratios in methanol (Scheme 2). We made minor experimental adjustments based on a previously reported method.^56^

**Figure 1:**
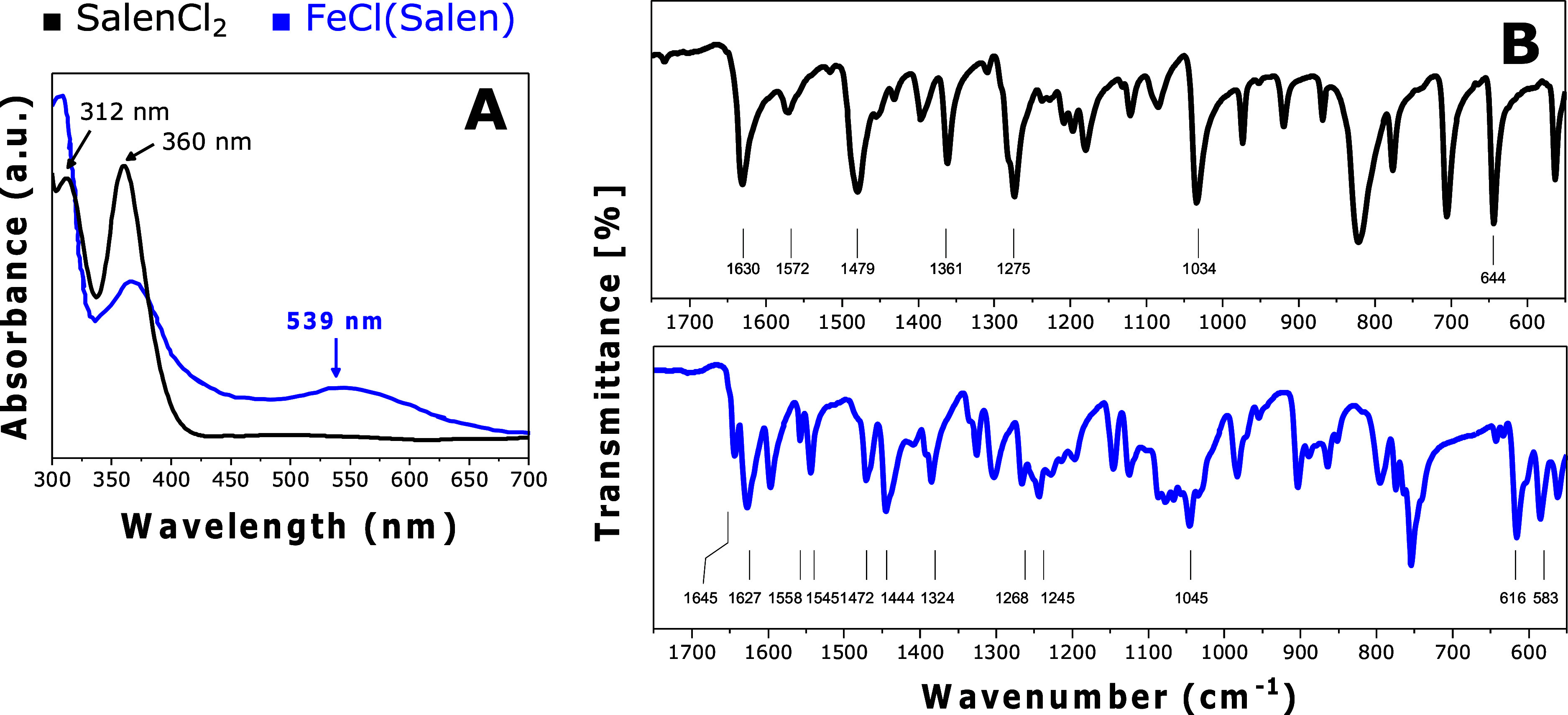
Preparation method of FeCl(Salen) with UV–vis and FT-IR ATR analysis. The two plots compare the UV–vis spectra in the CH_3_CN (A) and FT-IR 550–1750 cm^–1^ region (B) of the free ligand and iron complex.

In acetonitrile solution, the UV–vis spectrum (Figure 1A) of free SalenCl_2_ revealed two prominent absorption bands at 312 and 360 nm, corresponding to π → π* transition of the phenolic features. These bands were also observed in the spectrum of FeCl(Salen), for which a low-intensity absorption band at 539 nm was observed. This is attributed to the ligand-to-metal charge-transfer (LMCT) transition within the corresponding complex.^22,23^ To gain insight into the structural changes, we analyzed the IR spectra of the ligand and the complex (Figures 1B and S1). The SalenCl_2_ ligand exhibited a peak associated with C=N stretching at 1630 cm^–1^. In the corresponding complex, the peak shifted to 1627 cm^–1^, indicating C=N vibrational constraints due to metal coordination. C=O and C–O stretching bands were observed at 1572 and 1034 cm^–1^, respectively. Also, in this case, the presence of Fe causes modification in the C–O bonds’ vibrational transitions, leading to a shift of the peaks at lower wave numbers. Furthermore, the spectrum of the complex, in comparison to that of the free ligand, displayed a new peak at lower wavenumbers, around 583 cm^–1^, confirming the presence of the M–N bond.^22,23^

**Scheme 3:**
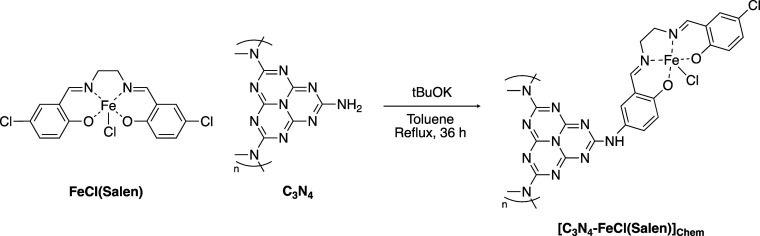
[C_3_N_4_-FeCl(Salen)]_Chem_ Hybrid Material Synthetic Strategy; the Hybrid Catalyst was Prepared Using a Coupling Reaction between Aryl Amines and Aryl Chlorides Catalyzed by Potassium *tert*-Butoxide

The hybrid material [C_3_N_4_-FeCl(Salen)]_Chem_ was synthesized via a coupling reaction, catalyzed by potassium *tert*-butoxide, between aryl amine and aryl chlorides (Scheme 3).^57,58^ In a Schlenk tube, C_3_N_4_, FeCl(Salen), and ^*t*^BuOK were suspended in dried toluene and refluxed for 36 h. The reaction was quenched with water, and the resulting solid was collected by centrifugation in DMSO, thoroughly washed with water, and subsequently dried at 80 °C overnight. An alternative hybrid material was prepared by physically mixing FeCl(Salen) with C_3_N_4_, designated as [C_3_N_4_-FeCl(Salen)]_Phys_.

**Figure 2:**
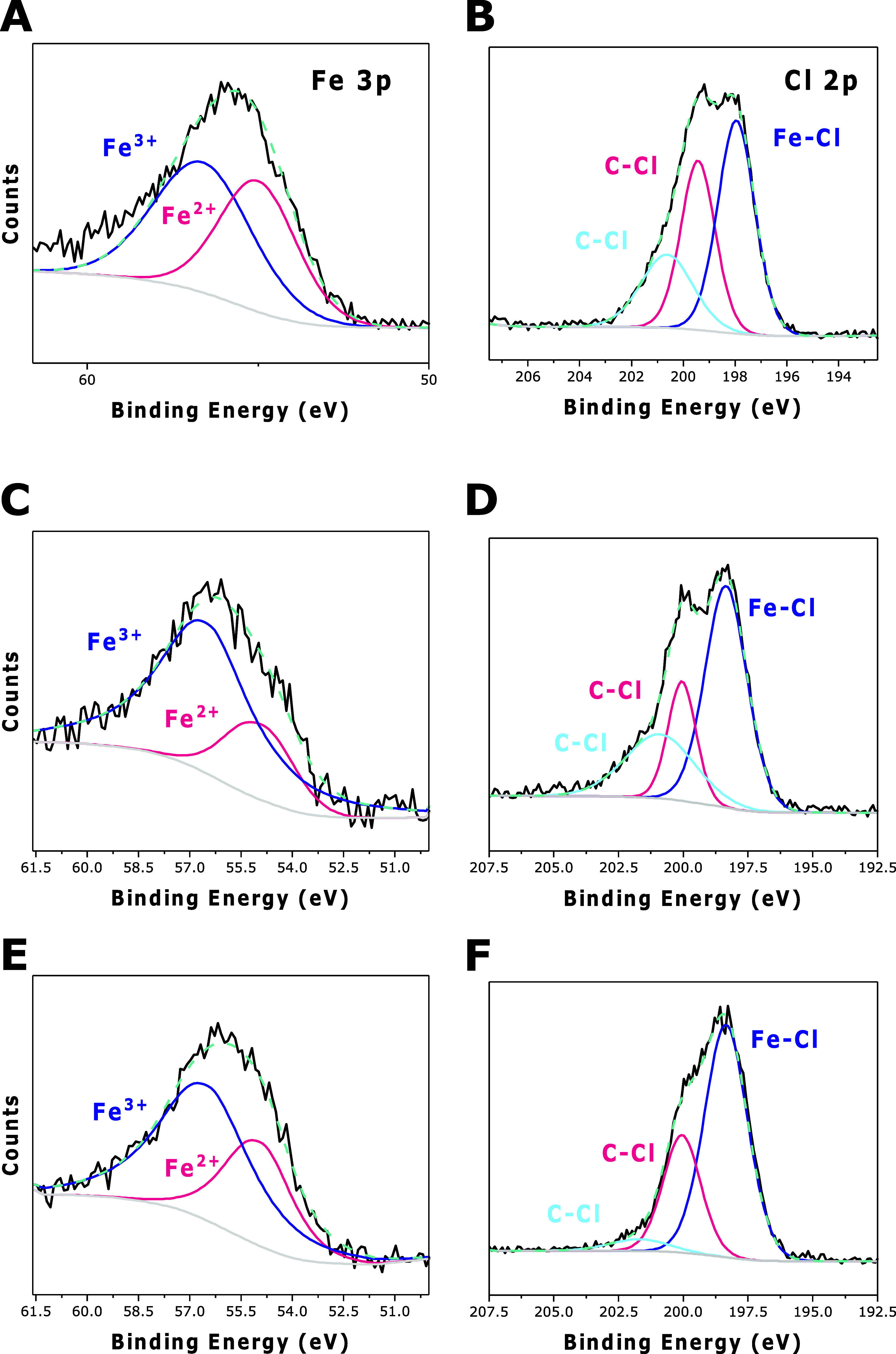
XPS analysis of FeSalenCl_2_, [C_3_N_4_-FeCl(Salen)]_Phys_ and [C_3_N_4_-FeCl(Salen)]_Chem_. XPS Spectra of Fe 3p and Cl 2p in FeSalenCl_2_ (A,B), [C_3_N_4_-FeCl(Salen)]_Phys_ (C,D), and [C_3_N_4_-FeCl(Salen)]_Chem_ (E,F).

The chemical states of the individual elements of [C_3_N_4_-FeCl(Salen)]_Phys_ and [C_3_N_4_-FeCl(Salen)]_Chem_ were investigated by using XPS. High-resolution XPS peaks of Fe 3p and Cl 2p and their corresponding best deconvolution to their different components are presented in Figure 2. The Fe 3p spectrum of [C_3_N_4_-FeCl(Salen)]_Phys_ shows a single peak that consists of both the Fe 3p_3/2_ and Fe 3p_1/2_ of Fe^n+^. The signals observed at 54.90 and 56.50 eV were assignable to Fe^2+^ and Fe^3+^, respectively (Figure 2A).^59^ Chloride 2p signals were observed at 200.05 and 200.90 eV, assignable to organic chloride bonds, and 198.35 eV to Fe–Cl (Figure 2B).^58^ The atomic abundance ratio between Fe and Cl is close to 1:3 (2.95), in agreement with the molecular structure, indicating a physical mixture of the two catalyst components (Table 1). A complete summary of the surface chemical composition from XPS deconvolution can be found in Table S1. The hybrid chemically bonded material [C_3_N_4_-FeCl(Salen)]_Chem_ displays clear signals for iron and chloride. Fe 3p signals were observed at 54.93 and 56.50 eV in close agreement with those of the pure complex and the physical mixture of FeCl(Salen) and C_3_N_4_ (Figure 2C). The chloride signal is weaker (Figure 2D) due to a loss in chloride content from the coupling reaction with amine groups of C_3_N_4,_ as illustrated in Scheme 3. The signal fits the three peaks, with a weaker intensity in the organic chloride presence, indicating the covalent interaction between the complex and C_3_N_4_. The atomic abundance ratio between iron and chloride is 1:2 (2.02), indicating a 35% loss of Cl bound to the Salen ligand, thus confirming that the coupling reaction has taken place.

**Table 1:** Selected At % Ratios^c^

At % ratio^a^	C_3_N_4_	FeSalenCl_2_	[C_3_N_4_-FeCl(Salen)]_Phys_	[C_3_N_4_-FeCl(Salen)]_Chem_
C/N^a^	0.69		1.05	1.21
Cl/Fe^a^		2.38	2.95	2.02
Cl/F^b^			2.38	1.56

a Calculated from XPS signals deconvolution.

b Calculated from ICP-MS and AQF-IC data for Fe and Cl, respectively.

c The “-” represents ratios that were not quantified as the signals were not observed or calculated.

A comprehensive study of the Cl/Fe ratios is shown in Table 1. The results were obtained using data from XPS, ICP-MS for iron content, and ion chromatography (AQF-IC) for chloride. All the techniques support the covalent bond formation, and the ratios were comparable to the theoretical ratios of 1:3 and 1:2 for [C_3_N_4_-FeCl(Salen)]_Phys_ and [C_3_N_4_-FeCl(Salen)]_Chem_, respectively.

The high-resolution XPS C 1s, N 1s, and O 1s spectra of C_3_N_4_, [C_3_N_4_-FeCl(Salen)]_Phys_, and [C_3_N_4_-FeCl(Salen)]_Chem_ are shown in Figure S2 in the Supporting Information. As can be seen, the graphitic support suffered negligible changes with complex interactions. The C 1s spectra show four components 285.03, 286.63, 288.21, and 289.01 eV assigned to sp C, C–N, N=C–N, and N–C–O/C–O, respectively, in all the samples.^36^ However, it is worth noting that the components assigned to carbon–nitrogen moieties have different contributions in the samples after C_3_N_4_ interaction with FeCl(Salen). The N 1s spectra show four components, 398.7, 399.5, 400.5, and 401.4 eV, which can be ascribed to pyridinic-N, primary, secondary, and tertiary amines, respectively. The presence of weaker signals for the primary amine contribution in [C_3_N_4_-FeCl(Salen)]_Chem_ was attributed to the loss in amino-terminal groups after the coupling reaction. Finally, O 1s spectra show four components after the interaction with FeCl(Salen) attributed to C–O, OH, and Fe–O and Fe–OH bonds. Table S1 summarizes the surface chemical composition of the samples.

**Figure 3:**
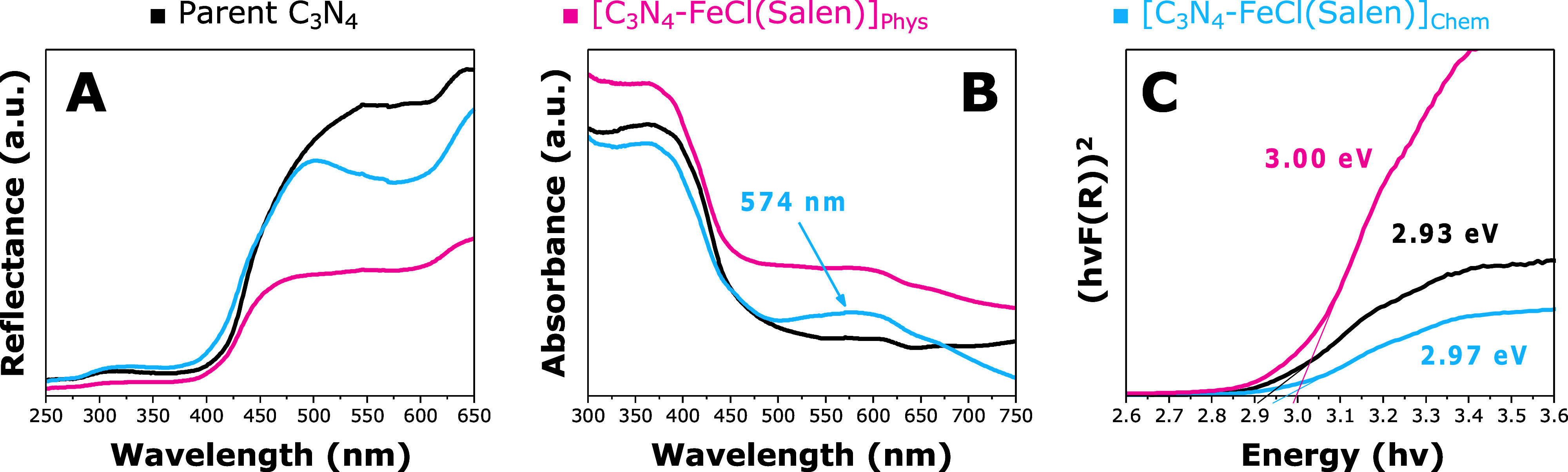
UV–vis spectroscopy characterization of C_3_N_4_, [C_3_N_4_-FeCl(Salen)]_Phys_, and [C_3_N_4_-FeCl(Salen)]_Chem_. The plots show the optical and structural features of the target [C_3_N_4_-FeCl(Salen)]_Chem_ compared with the parent graphitic carbon nitride and the nanocomposite prepared via the physical method, [C_3_N_4_-FeCl(Salen)]_Phys_. The plots of the figure are related to UV–vis absolute reflectance spectra (A), UV–vis absorbance spectra (B), and calculated Tauc plots with the corresponding energy band gap values (C).

The hybrid [C_3_N_4_–FeCl(Salen)]_Chem_ was then characterized using diffuse reflectance UV–vis spectroscopy alongside [C_3_N_4_-FeCl(Salen)]_Phys_ and pristine C_3_N_4_ (Figure 3). The comparison of the three samples revealed a new absorption peak at 574 nm and increased absorption throughout the visible range, attributable to the presence of the FeCl(Salen) complex (Figure 3B). The retention of the LMCT band in [C_3_N_4_-FeCl(Salen)]_Chem_ indicates the preservation of the molecular structure postreaction with C_3_N_4_. The red-shift in the absorption maximum of the immobilized FeCl(Salen) from 539 to 574 nm when compared with the complex in solution can be attributed to a larger conjugation of the hybrid material. The bandgaps, as determined from the Tauc plots (Figure 3C),^60^ were found to be 2.93 eV for C_3_N_4_, 3.00 eV for [C_3_N_4_-FeCl(Salen)]_Phys_, and 2.97 eV for [C_3_N_4_-FeCl(Salen)]_Chem_. The measured energy band gap of graphitic carbon nitride is in good agreement with the values reported in the literature.^36,61^ The presence of the complex widens the gap between the valence and conduction bands of the materials and the parent C_3_N_4_.^37,62,63^

**Figure 4:**
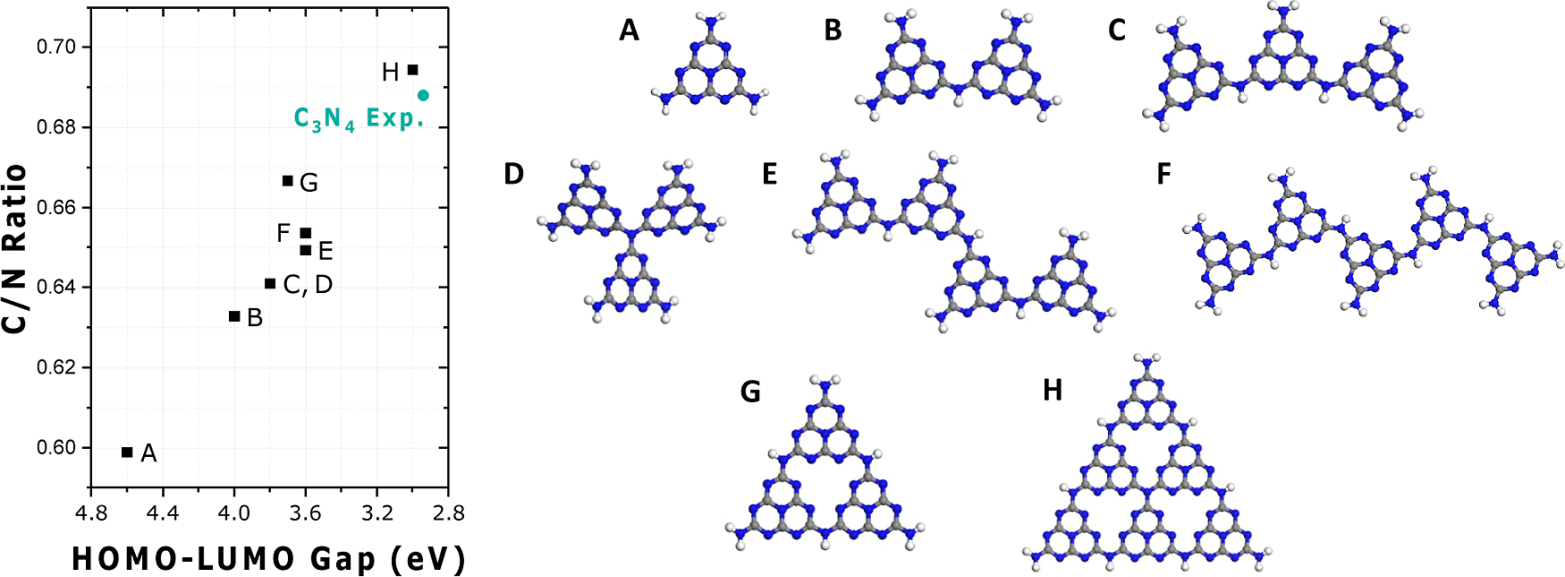
Calculated HOMO–LUMO gap vs C_3_N_4_ structures with varying sizes (HSE06). The data points were labeled according to the optimized configurations (A–H) shown and plotted in comparison with the experimental C_3_N_4_ (named “C_3_N_4_ Exp.” in the graph). Gray, blue, and white spheres represent C, N, and H, respectively.

DFT calculations were performed to examine how the highest occupied molecular orbital (HOMO)–lowest unoccupied molecular orbital (LUMO) gap changes with the C_3_N_4_ size and correlates with the immobilized complex on its surface. First, the size effect of pure C_3_N_4_ was studied with different repeated units of the heptazine ring (melem units) and C/N ratio. Figure 4 shows that the HOMO–LUMO gap decreases with an increased C/N ratio and melem units due to the larger conjugation. G and H models are representative of C_3_N_4_ with defects, not idealized structures, similar to what was observed experimentally, as shown in Figure 3C (Figure S3).

**Figure 5:**
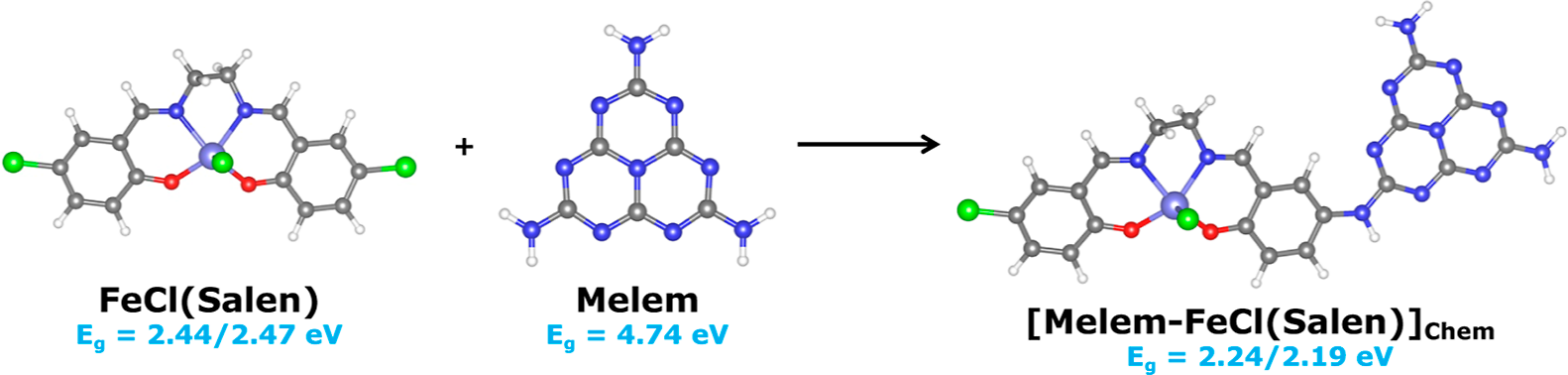
Calculated HOMO–LUMO gaps FeCl(Salen), Melem, and [Melem-FeCl(Salen)]Chem (HSE06). The energy band gap (*E*_g_) was studied for both high- and low-spin (left and right values, respectively) optimized geometries of the metal complex. Gray, blue, and white spheres represent C, N, and H, while green, red and purple represent Cl, O, and Fe.

The experimental band gap of C_3_N_4_ is comparable with the structure labeled as H, as expected. Instead, the HOMO–LUMO gap of the hybrid [C_3_N_4_-FeCl(Salen)]_Chem_ was evaluated by studying the configuration melem-complex (Figure 5). The optimized geometry of Melem, FeCl(Salen), and [Melem-FeCl(Salen)]_Chem_ was calculated and is shown in Figure S4. The electronic structures of FeCl(Salen) and [Melem-FeCl(Salen)]_Chem_ were studied for both high- and low-spin-optimized geometries of the metal complex. This is due to the oxidation state of the iron(III) in the FeCl(Salen) complex. The DFT results are shown in Figure 5 and are summarized in Table S2. The predicted band gaps from the DFT model of [Melem-FeCl(Salen)]_Chem_ are closer to those of FeCl(Salen) due to the conjugation constraints from the single Melem unit.

DFT was also used to calculate the UV–vis spectra of [Melem-FeCl(Salen)]_Chem_ (Table S3). The results validated the presence of the feature at 574 nm, predicted at 572 nm, which was attributed to the presence of the FeCl(Salen) complex and its LMCT transition.

The (FT-IR ATR) spectra of pristine C_3_N_4_, [C_3_N_4_-FeCl(Salen)]_Phys_, and [C_3_N_4_-FeCl(Salen)]_Chem_ are presented in Figure S5. These IR patterns reveal nearly identical characteristics among the three materials, signifying minimal postfunctionalization changes in the bulk structure due to the low loading of the complex. The main observed peaks can be attributed to the graphitic carbon nitride support. For the parent C_3_N_4_, the broad asymmetric features observed between 2885 and 3250 cm^–1^ were attributed to N–H sites, bridging –NH– and –NH_2_ terminal groups. A large number of relatively sharp peaks extending throughout the 700–1700 cm^–1^ were assigned to C–N and NCN-CNC bending vibrations.^36^ A sharp peak occurring at 804 cm^–1^ was assigned to the heptazine motifs, according to the literature.^64^ Features below 620 cm^–1^ might be due to N–H deformation vibrations.^65^ However, in the case of the covalently functionalized materials, either new peaks appear or old ones are shifted, indicating the introduction of new interactions on the C_3_N_4_ moiety. For example, in the case of the covalently bonded ligand to C_3_N_4_, labeled as C_3_N_4_-(SalenCl_2_), a new feature at 3682 cm^–1^ was assigned to the phenolic vibrations of the salen-type ligand. Moreover, in the C–N vibrations region of [C_3_N_4_-FeCl(Salen)]_Phys_, and [C_3_N_4_-FeCl(Salen)]_Chem_, a relatively small shift of several peaks was attributed to the constraints introduced by the ligand and the complex on the parent moiety. Similar phenomena were already observed in other studies on C_3_N_4_ as support for molecular catalysts.^49,66^

**Figure 6:**
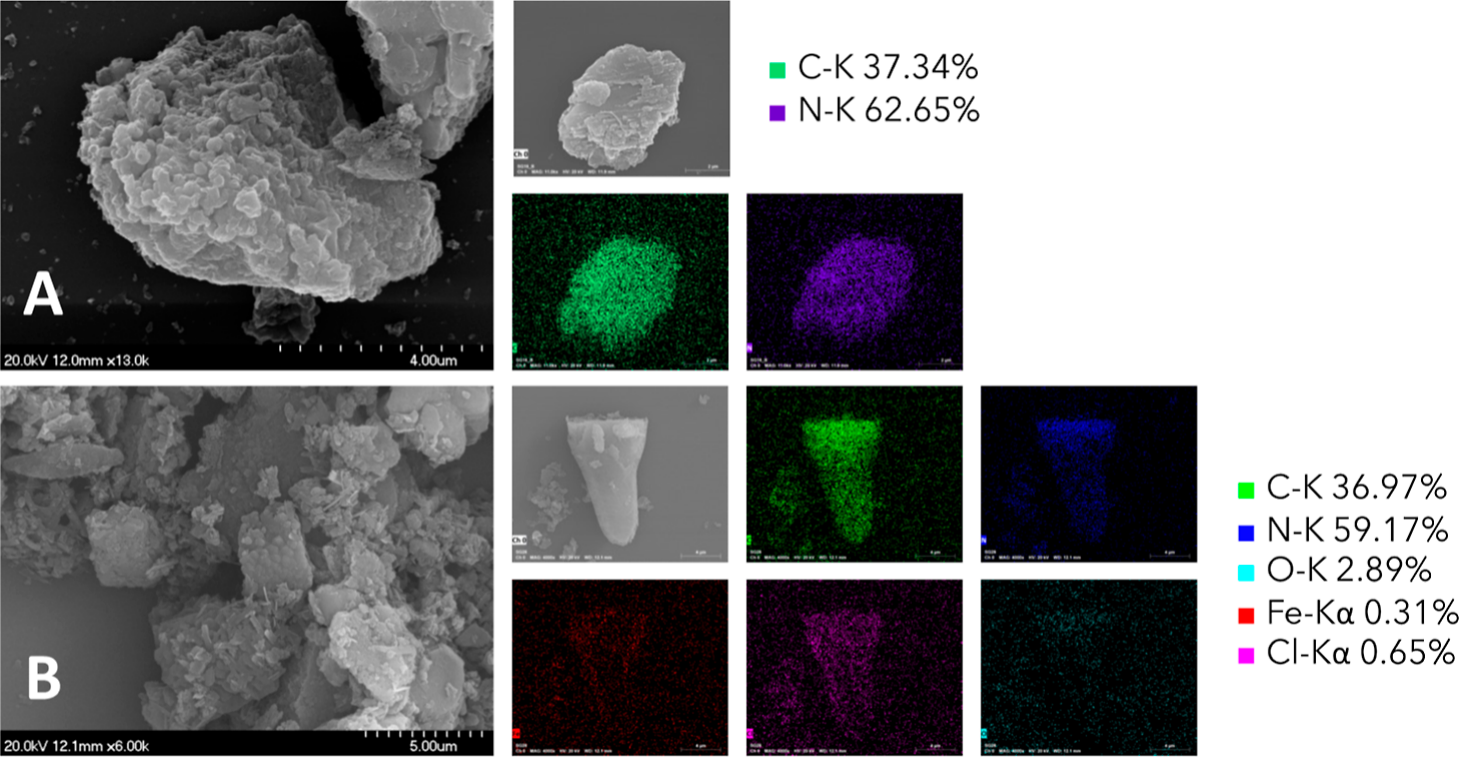
Microscopy characterization of the C_3_N_4_ and [C_3_N_4_-FeCl(Salen)]_Chem_ samples. SEM pictures and EDX elemental mapping of C_3_N_4_ (A) and [C_3_N_4_-FeCl(Salen)]_Chem_ (B).

SEM/EDX images, as shown in Figures 6 and S6, were captured to elucidate the morphology and elemental distribution of the examined samples. When compared with the pristine C_3_N_4_ (Figure 6A), [C_3_N_4_-FeCl(Salen)]_Chem_ (Figure 6B) not only retained its original structure but also exhibited crumpling features attributed to the specific reaction conditions employed for the binding of the complex to the C_3_N_4_ surface. In contrast, [C_3_N_4_-FeCl(Salen)]_Phys_, representing the physical mixture of the two compounds, displayed distinct characteristics. It was possible to distinguish two predominant morphologies assigned to the iron complex (Figure S6C,D) and the supporting C_3_N_4_ (Figure S6A), respectively. These results were confirmed with EDX scans within the two morphologies.

The SEM/EDX elemental mapping of [C_3_N_4_-FeCl(Salen)]_Chem_ (Figures 6B and S6B) does not suggest the formation of large iron agglomerates. This observation underscores the effectiveness of the binding process and the intimate interaction between the complex and the C_3_N_4_ support.

### 3.2 Photocatalytic Epoxidation of Olefins Screening and Stability

**Figure 7:**
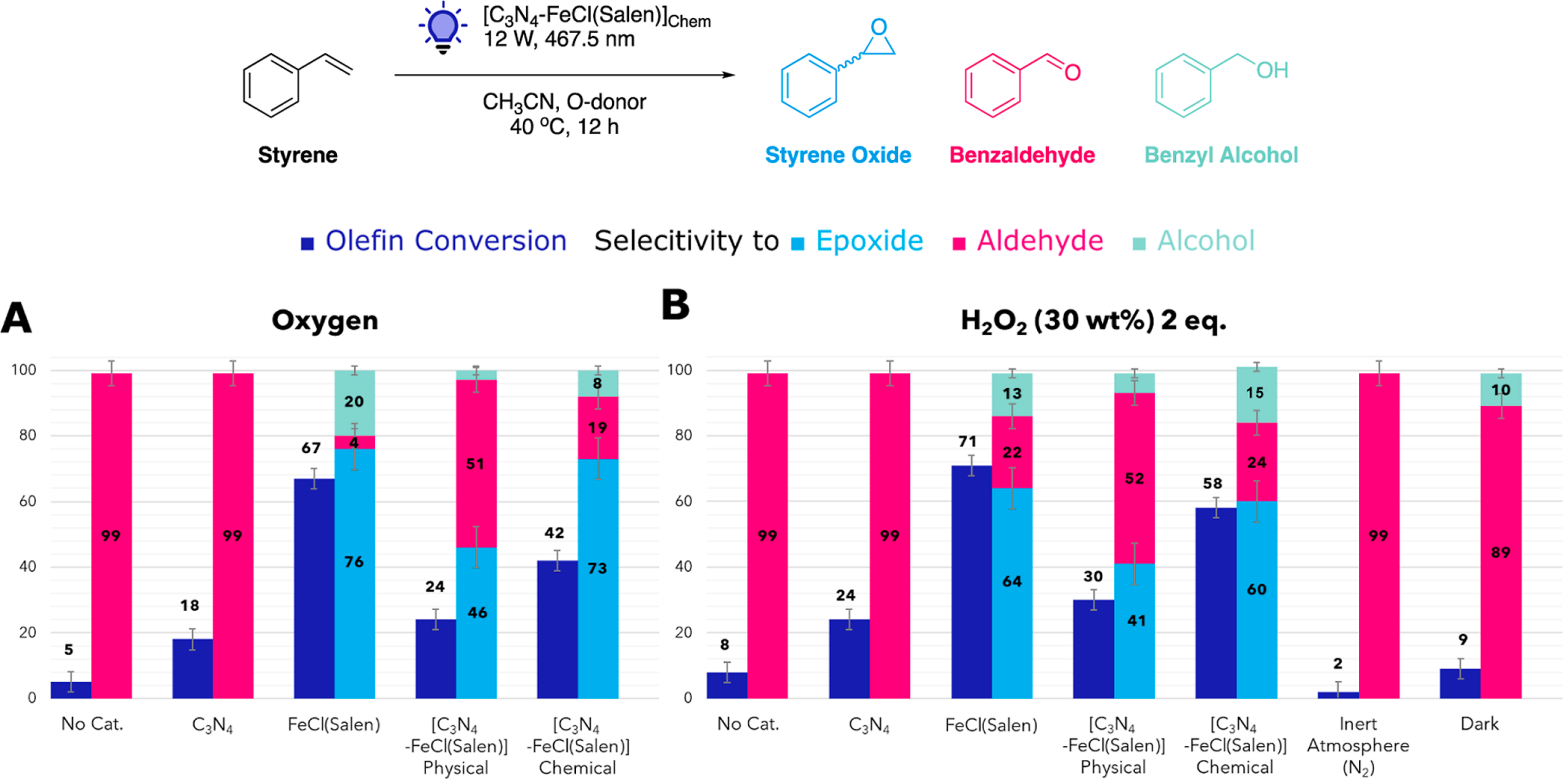
Preliminary studies and single-factor tests for the photocatalytic epoxidation of styrene. Preliminary studies and distribution of oxidation products of styrene epoxidation were assessed using C_3_N_4_, FeCl(Salen), [C_3_N_4_-FeCl(Salen)]_Phys_, and [C_3_N_4_-FeCl(Salen)]_Chem_. The performance was evaluated using oxygen (A) and hydrogen peroxide 30 wt % (B) as O-donor species. Reaction conditions for test A: styrene (1 mmol), CH_3_CN (5 mL), catalyst (5 mg or 0.083 mg/mL for homogeneous catalyst), and O_2_ (balloon), stirring for 12 h under illumination (blue LED, 467.5 nm, 12 W). Reaction conditions for test B: styrene (1 mmol), CH_3_CN (5 mL), catalyst (5 mg or 0.083 mg/mL for homogeneous catalyst), and H_2_O_2_ 30 wt % (2 equiv), stirring for 12 h under illumination (blue LED, 467.5 nm, 12 W).

After successfully preparing [C_3_N_4_-FeCl(Salen)]_Chem_, its catalytic performance was evaluated by conducting epoxidation of olefins. We first studied the oxidation of styrene in the presence of pure gaseous oxygen by exposing a suspension of the catalyst with the olefin to blue LED for 12 h with an oxygen balloon. The catalyst screening results are summarized in Figure 7 and Table S4. The identified products of the reaction were aldehydes, alcohols, and epoxides. Styrene conversion and selectivity toward styrene oxide were retained after covalent anchoring of FeCl(Salen) to C_3_N_4_ (Figure 7A, [C_3_N_4_-FeCl(Salen)] Chemical). Control experiments were conducted to crosscheck the activity of the hybrid material. In the absence of C_3_N_4_ and FeCl(Salen), traces of benzaldehyde were observed, while C_3_N_4_ catalyzed the reaction toward benzaldehyde with low conversion. The physical mixture of the complex and C_3_N_4_ successfully catalyzed the reaction with poor conversion and low selectivity (Figure 7A, [C_3_N_4_-FeCl(Salen)] Physical).

Next, the oxidant effect was investigated by switching to hydrogen peroxide (H_2_O_2_), a polar species completely miscible in acetonitrile, in contrast with diatomic oxygen (Figure 7B). In this case, the conversion of styrene was higher than that with O_2_. This could be attributed to the competitive oxygen reduction reaction to H_2_O_2_ in the presence of gaseous O_2_ by surface-modified carbon nitrides, in agreement with previously reported results.^67^ Furthermore, H_2_O_2_ has a higher oxidation potential than O_2_, which results in higher oxidative properties and reactivity.^68^ Conversely, the selectivity toward styrene oxide was lower across all the samples. This was associated with the reactivity of H_2_O_2_ and the formation of the hydroxide radical, which induces the olefin bond break and overoxidation of styrene.^69,70^ Control experiments showed that no epoxide was formed if the reaction was conducted without a catalyst (No Cat.), without an oxidizing agent (Inert Atmosphere), or under dark conditions (Dark). (Figure 7A,B).

The activity of a photocatalyst is dependent not only on the metal amount and its dispersion on the support but also on the availability of the catalyst during the reaction and its suspension in the reaction mixture.^47^ Therefore, the solvent effect was also investigated by using hydrogen peroxide as the oxidizing agent (Figure S7). The amino-terminal groups of C_3_N_4_ promote homogeneous suspension in solvents with higher polarity, and the nitrogen atoms in acetonitrile effectively interact with the C–N framework of C_3_N_4,_ facilitating contact with the organic substrate.^28,66^ Overall, [C_3_N_4_-FeCl(Salen)]_Chem_ demonstrated photocatalytic selectivity comparable to that of the free complex in aerobic oxidation of styrene, in accordance with the already reported results on epoxidation photocatalyzed by carbon nitride.^67^ The conversion was improved with the use of H_2_O_2_ to the detriment of selectivity. Control experiments were performed, and the results are shown in Figure 7A,B. No epoxide was detected if the reaction was conducted without a catalyst (No Cat.), without an oxidizing agent (Inert Atmosphere), and under dark conditions (Dark). Overall, [C_3_N_4_-FeCl(Salen)]_Chem_ demonstrated photocatalytic selectivity comparable to that of the free complex in aerobic oxidation of styrene, in accordance with already reported results on epoxidation photocatalyzed by carbon nitride.^67^ The conversion was improved with the use of H_2_O_2_ to the detriment of selectivity.

**Figure 8:**
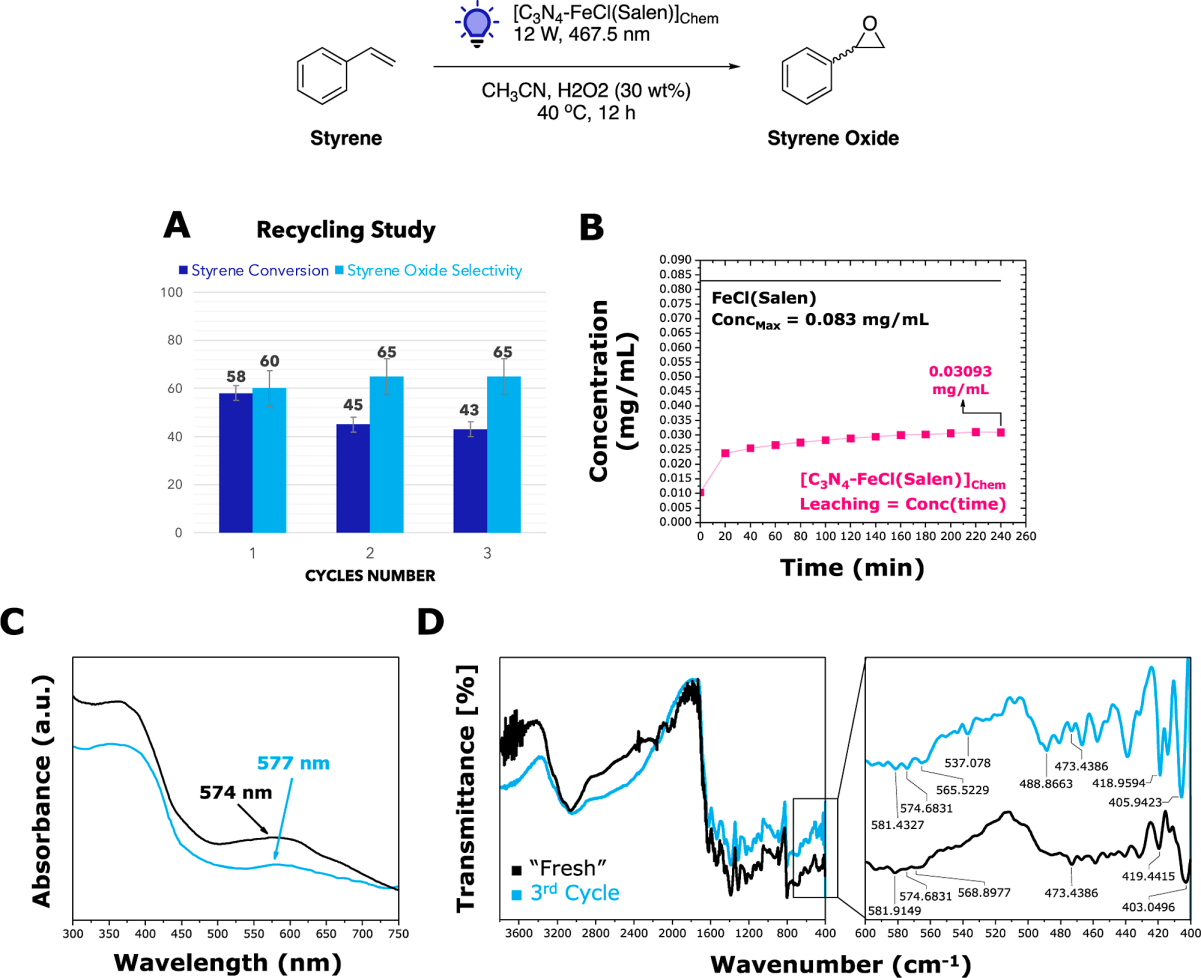
Catalyst recycling and complex leaching studies for styrene epoxidation. The reaction conditions of the recycling studies (A) were 1 mmol of styrene, 5 mL of CH_3_CN, 5 mg of catalyst, and two equivalents of H_2_O_2_ 30 wt %. The slurry mixture was stirred for 12 h, and the catalyst was recovered via centrifugation, washed with water and CH_3_CN, and dried overnight at 80 °C. The complex leaching study (B) was performed using the same reaction conditions (for a shorter time, 4 h) using in situ UV–vis spectroscopy. The concentration vs time plot was obtained from standard UV–vis curves of complex solutions and fixed concentrations and by monitoring the absolute absorbance as a function of time. The catalyst was tested in three consecutive cycles (A) and was characterized by UV–vis diffuse reflectance spectroscopy (C) and FT-IR KBr (D) after the third cycle.

The importance of catalyst reusability cannot be overstated, as it contributes significantly to the activity and availability of the catalyst during the reaction, influencing the process performance and cost-effectiveness. Following photocatalytic epoxidation, the catalyst was efficiently recovered from the slurry mixture through centrifugation and found to be ready for recycling after a simple washing step involving water and acetonitrile. Notably, the conversion of styrene exhibited a slight decrease after the initial use, while the catalyst’s selectivity improved, reaching up to 65% (Figure 8A). We conducted a leaching test (Figure 8B) by monitoring the absorption peaks of the slurry mixture every 20 min with UV–vis spectroscopy. These intensities were compared with standard curves derived from complex solutions at varying concentrations (Figure S8). Overall, 62.7% (estimated 0.123 mg in 5 mg of material) of the complex was still anchored to the C_3_N_4_ surface after 4 h before reaching a plateau. The result was also validated by DFT calculations of the [C_3_N_4_-FeCl(Salen)]_Chem_ formation energy. The calculated values of −10.7 kJ·mol^–1^ (low spin) and −12.1 kJ·mol^–1^ (high spin) indicate an energetically favorable reaction. However, such relatively higher formation energy suggests that the hybrid material may not be very stable.

To understand the stability of the complex, we subjected the same catalysts to three consecutive cycles and analyzed them via UV–vis diffuse reflectance spectroscopy (Figure 8C) and FT-IR KBr (Figure 8D). The results confirmed that the material’s framework remained unchanged after multiple runs (Figure 8C). This observation underscores the recyclability of [C_3_N_4_-FeCl(Salen)]_Chem_ for practical catalytic applications and further research to improve the binding of molecular complexes onto the C_3_N_4_ surface.

The catalytic performance of [C_3_N_4_-FeCl(Salen)]_Chem_ was evaluated under aerobic conditions for different olefin substrates, such as cyclohexene, α-pinene, 1-octene, and *cis*-4-octene. The results are summarized in Table 2. The product distributions of the different entries are shown in Table S5. The catalyst showed high selectivity and good conversion in the epoxidation of cyclohexene and linear olefins, with the corresponding epoxides as the primary product. Oxidation of α-pinene showed overall selectivity toward the epoxide. However, the reduction in the yield can be attributed to the decomposition and rearrangement of the epoxide due to the dipole moment of C–O and its angle strain, resulting in the formation of a significant amount of byproducts.^71^

**Table 2:**
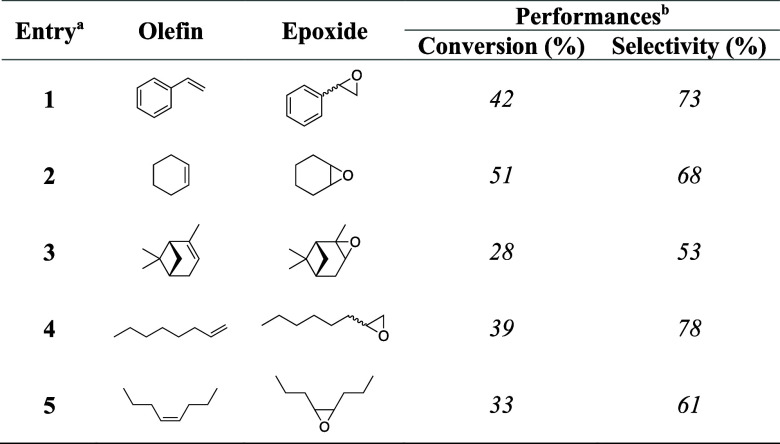
Substrate Scope of Aerobic Photocatalytic Epoxidation Using [C_3_N_4_-FeCl(Salen)]_Chem_

a Reaction conditions: olefin (1 mmol), CH_3_CN (5 mL, solvent), catalyst (5 mg), and O_2_ (balloon). The mixture was continuously stirred under illumination (blue LED, 467.5 nm, 12 W) for 12 h.

b Calibrated conversion and selectivity determined by GC.

## 4 Conclusions

Our research focused on developing a novel catalytic system, [C_3_N_4_-FeCl(Salen)]_Chem_, for the photocatalytic epoxidation of various olefins. This hybrid catalyst, synthesized by anchoring iron salen-type complexes onto graphitic carbon nitride (C_3_N_4_), demonstrated promising catalytic performance in terms of the selectivity for epoxide formation and ease of recyclability. It exhibited cyclic and linear olefin activity, including cyclohexene, α-pinene, 1-octene, and *cis*-4-octene.

Our approach leverages the intrinsic amino-terminal groups within C_3_N_4_ as anchoring points, eliminating the need for additional linkers or the formation of heterojunctions with other semiconductors. This work marks the first reported instance of this material, and its ease of preparation and reusability are significant steps forward in developing environmentally friendly epoxidation techniques. However, it is important to note that while our catalyst shows promising performance and recyclability, it may not be the most active or stable option available. Further research is needed to optimize the anchoring strategy and improve the overall performance.

## Supporting Information Available

Further materials characterization data (XPS, FT-IR, SEM/EDX, and UV–vis), experimental and computational (HOMO–LUMO levels, energy band-gaps, UV–vis, and geometries) (PDF)
The results are available free of charge in the Supporting Information at https://pubs.acs.org/doi/XXX
