# Supplementary material for: Covalently Anchored Molecular Catalyst onto a Graphitic Carbon Nitride Surface for Photocatalytic Epoxidation of Olefins
Source: ACS Catal. 2024 Sep 18;14(19):14639–51. doi: 10.1021/acscatal.4c04187 (PMC11459433; doi:10.1021/acscatal.4c04187)
Supplement: Supplementary file 1 — cs4c04187_si_001.pdf [file cs4c04187_si_001.pdf]

SUPPLEMENTAL INFORMATION

Covalently Anchored Molecular Catalyst onto Graphitic Carbon Nitride Surface for Photocatalytic Epoxidation of Olefins

Sebastiano Gadolini,[1,] Rachel N. Kerber,[1] Riho T. Seljamäe-Green,[1] Wenming Tong,[2] Pau Farràs,[2,*] and Elena C. Corbos.[1]*

[1]Johnson Matthey Technology Centre, Blounts Court, Sonning Common, Reading RG4 9NH, United Kingdom.

[2]School of Biological and Chemical Sciences, Energy Research Centre, Ryan Institute, University of Galway, University Road, Galway H91 CF50, Ireland

Sebastiano Gadolini – *Johnson Matthey Technology Centre, Blounts Court, Sonning Common, Reading RG4 9NH, United Kingdom. Email: sebastianogadolini@outlook.com*

Pau Farràs – *School of Biological and Chemical Sciences, Energy Research Centre, Ryan Institute, University of Galway, University Road, Galway H91 CF50, Ireland. Email: pau.farras@universityofgalway.ie*

Table S1. Surface Chemical Composition (XPS)

Element (At%)	C_3N_4	$FeSalenCl_2$	[C_3N_4-FeCl(Salen)]$_{Phys}$	[C_3N_4-FeCl(Salen)]$_{Chem}$
Carbon	39.67	69.23	45.23	49.36
Nitrogen	59.65	1.79	42.88	40.79
Oxygen	0.69	20.84	3.91	3.84
Chlorine	-	5.74	5.97	4.02
Iron	-	2.41	2.02	1.99

Table S2. HOMO-LUMO levels and energy band-gaps (eV) of FeCl(Salen), and [Melem-FeCl(Salen)]$_{Chem}$

Species	HOMO	LUMO	E_g (eV)
Melem	-6.199	-1.462	4.738
FeCl(Salen) Low Spin	-5.751	-3.311	2.440
FeCl(Salen) High Spin	-5.918	-3.449	2.469
[Melem-FeCl(Salen)]$_{Chem}$ Low Spin	-5.384	-3.140	2.243
[Melem-FeCl(Salen)]$_{Chem}$ High Spin	-5.463	-3.274	2.189

Table S3. Calculated UV-vis of [Melem-FeCl(Salen)]$_{Chem}$

Excitation (Fe High Spin)	Energy (nm)	Oscillation (Velocity)	Oscillation (Length)
1	571.848	0.00054	0.0005
2	499.559	0.01171	0.01247
3	489.936	0.0151	0.01597
4	466.402	0.00986	0.00998
5	455.468	0.0854	0.08927
6	439.023	0.02487	0.02684
7	427.756	0.00463	0.00478
8	413.885	0.00498	0.00496
9	404.716	0.01181	0.01219
10	395.044	0.01379	0.01498

Table S4. Preliminary studies and single-factor tests epoxidation of styrene summary

Entry	Catalyst	Oxidizing Agent	Conversion (%)[c]	Selectivity (%)[c]		
				Epoxide	Aldehyde	Alcohol
1	-	O_2	5	-	99	-
2	C_3N_4	O_2	18	-	99	-
3	FeCl(Salen)	O_2	67	76	4	20
4	[C_3N_4-FeCl(Salen)]$_{Phys}$	O_2	24	46	51	3
5	[C_3N_4-FeCl(Salen)]$_{Chem}$	O_2	42	73	19	8
6	-	H_2O_2 (30 wt%)	8	-	99	-
7	C_3N_4	H_2O_2 (30 wt%)	24	-	99	-
8	FeCl(Salen)	H_2O_2 (30 wt%)	71	64	22	13

**9**	$[C_3N_4\text{-FeCl(Salen)}]_{Phys}$	H_2O_2 (30 wt%)	30	41	52	6
**10**	$[C_3N_4\text{-FeCl(Salen)}]_{Chem}$	H_2O_2 (30 wt%)	58	60	24	15
**11**[a]	$[C_3N_4\text{-FeCl(Salen)}]_{Chem}$	N_2	2	2	99	-
**12**[b]	$[C_3N_4\text{-FeCl(Salen)}]_{Chem}$	H_2O_2 (30 wt%)	9	9	89	10

[a]Reaction conditions: olefin (1 mmol), CH_3CN (5 mL, solvent), catalyst (5 mg), and N_2 (balloon). The mixture was continuously stirred under illumination (blue LED, 467.5 nm, 12 W) for 12 hours in inert conditions.
[b]Entry 12 was performed in dark conditions.
[c]Calibrated conversion and selectivity determined by GC.

Table S5. Aerobic photocatalytic epoxidation scope of the reaction catalyzed by $[C_3N_4\text{-FeCl(Salen)}]_{Chem}$

Entry[a]	Olefin	Conversion (%)[b]	Selectivity (%)[b]		
			Epoxide	Aldehyde	Alcohol
1	Styrene	42	73	19	8
2	Cyclohexene	51	68	18	14
3	α-Pinene	28	53	36	11
4	1-Octene	39	78	12	10
5	*cis*-4-Octene	33	61	22	17

[a]Reaction conditions: olefin (1 mmol), CH_3CN (5 mL, solvent), catalyst (5 mg), and O_2 (balloon). The mixture was continuously stirred under illumination (blue LED, 467.5 nm, 12 W) for 12 hours.
[b]Calibrated conversion and selectivity determined by GC.

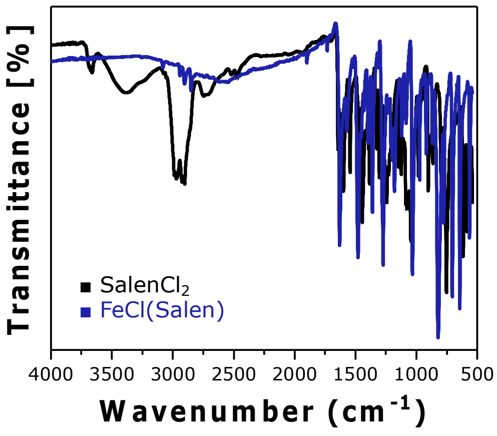

Figure SI 1. FT-IR ATR spectra of $SalenCl_2$ and FeCl(Salen). Stacked graph of the zoomed FT-IR ATR spectra of the free ligand and the corresponding iron complex in transmittance mode.

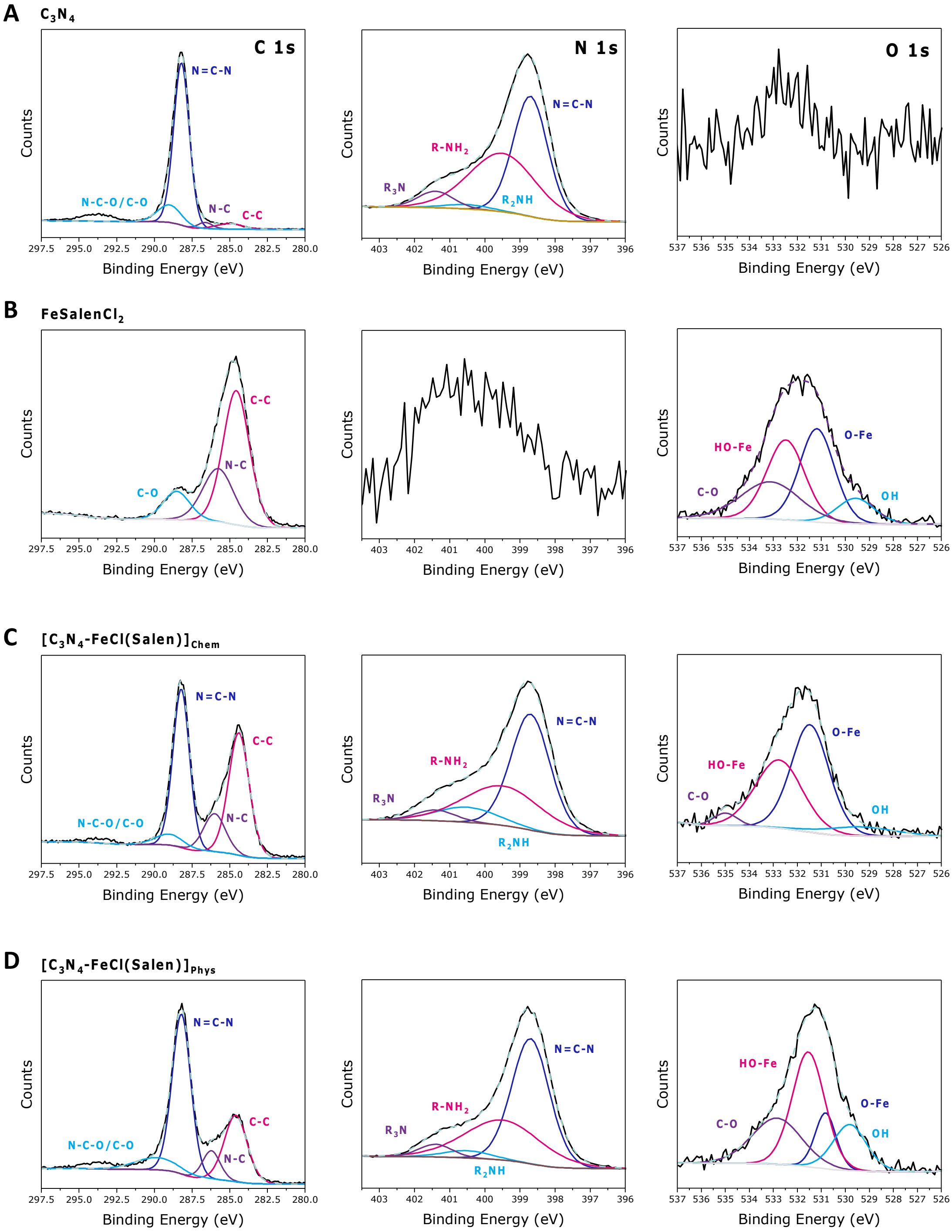

Figure S2. XPS analysis of C_3N_4, $FeSalenCl_2$, $[C_3N_4\text{-FeCl(Salen)}]_{Phys}$ and $[C_3N_4\text{-FeCl(Salen)}]_{Chem}$. XPS Spectra of C 1s, N 1s, and O 1s of C_3N_4, $FeSalenCl_2$, $[C_3N_4\text{-FeCl(Salen)}]_{Phys}$, and $[C_3N_4\text{-FeCl(Salen)}]_{Chem}$, A-D respectively.

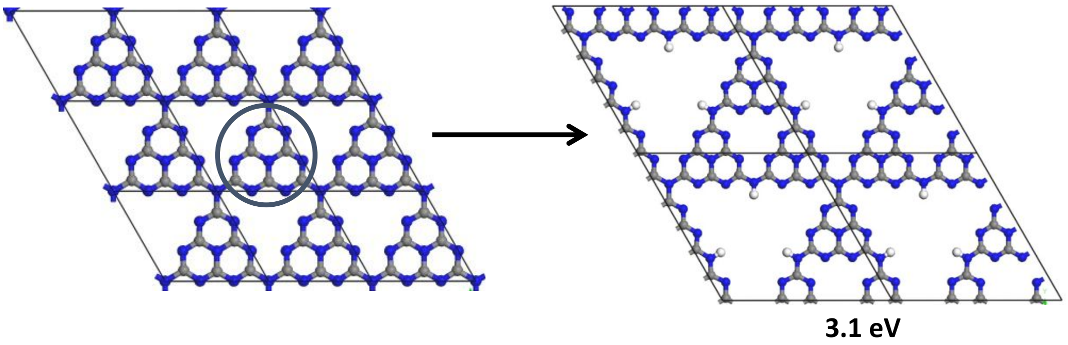

Figure S3. Periodic structure of C_3N_4 with melem defects (HSE06).

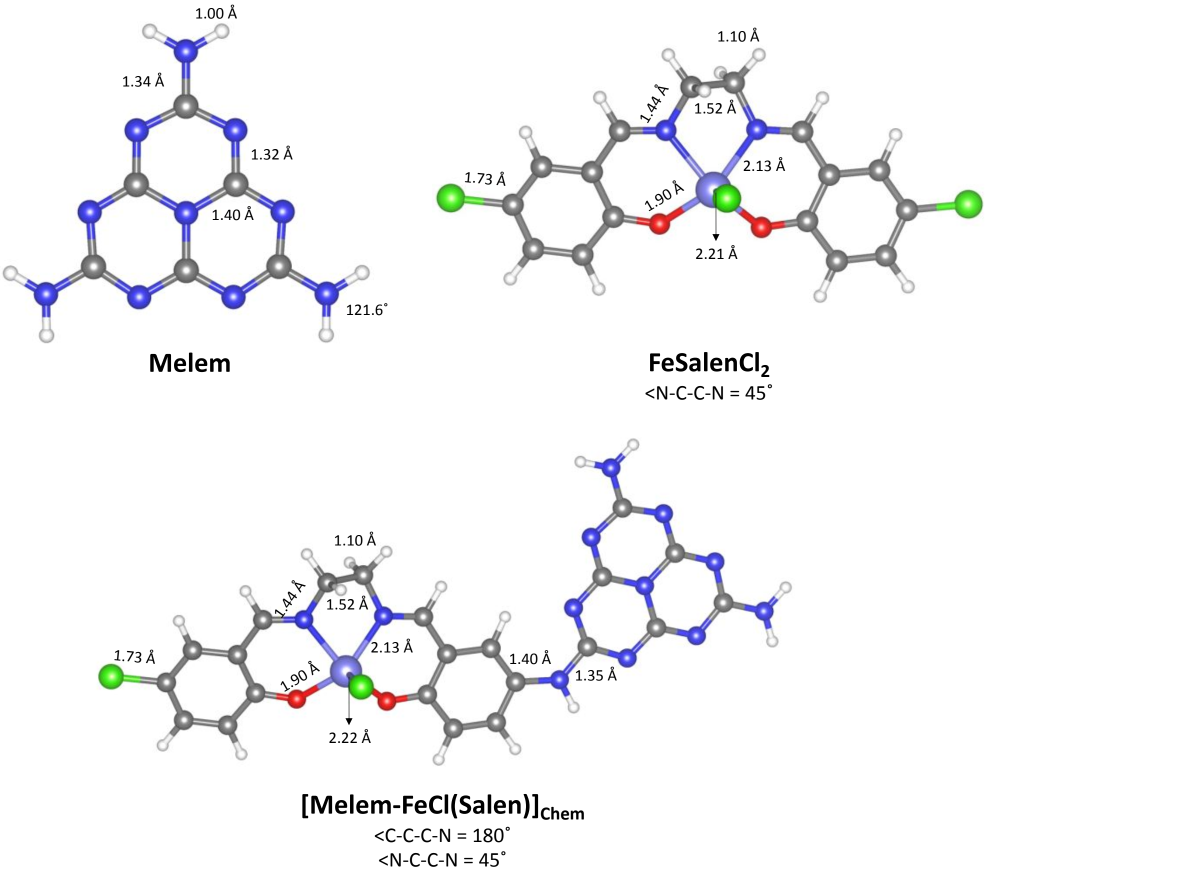

Figure S4. Optimized geometries for Melem, FeCl(Salen) and $[\text{Melem-FeCl(Salen)}]_{\text{Chem}}$ structure (HSE06). Iron geometries were optimized for high-spin complex.

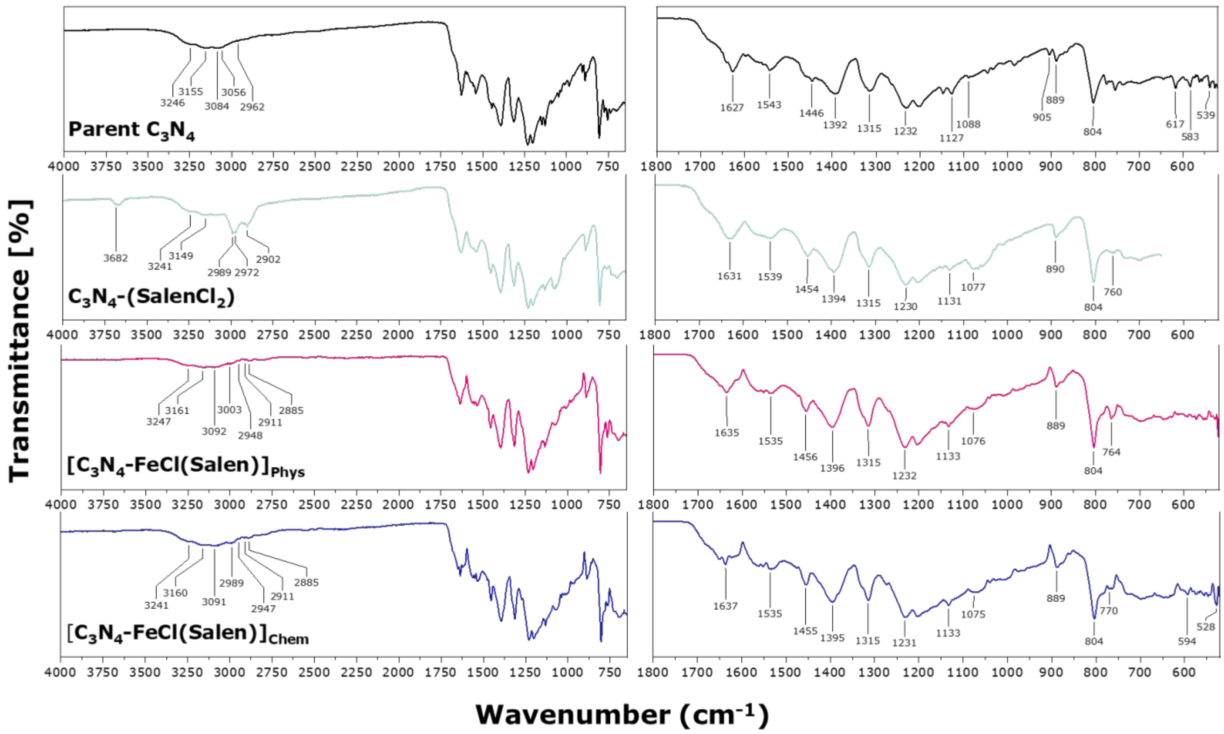

Figure S5. FT-IR ATR spectra of the parent C_3N_4, C_3N_4-($SalenCl_2$), [C_3N_4-FeCl(Salen)]$_{Phys}$ and [C_3N_4-FeCl(Salen)]$_{Chem}$. Stacked graph of the overall and zoomed (1800-550 cm^{-1}) FT-IR ATR spectra of the supporting C_3N_4, of the C_3N_4 functionalized with the Schiff base C_3N_4-($SalenCl_2$), and the nanocomposites C_3N_4-iron complex prepared by physical mixture [C_3N_4-FeCl(Salen)]$_{Phys}$ and covalent interaction [C_3N_4-FeCl(Salen)]$_{Chem}$, respectively. The spectra show that the main peaks can be attributed to the graphitic carbon nitride support. However, in the case of the covalently functionalized materials, either new peaks appear or are shifted, indicating the introduction of new interactions on the C_3N_4 moiety.

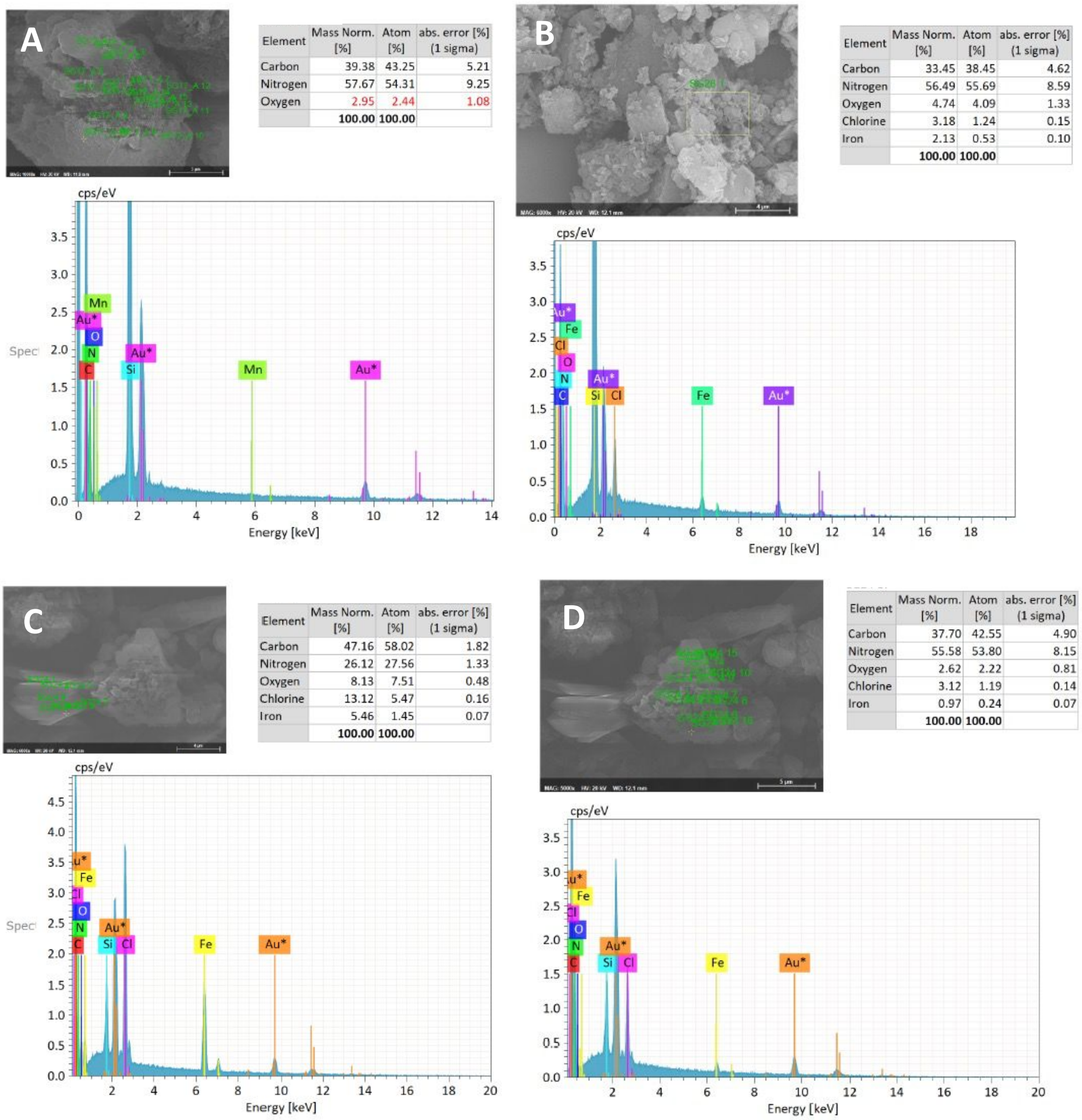

A

Element	Mass Norm. [%]	Atom [%]	abs. error [%] (1 sigma)
Carbon	39.38	43.25	5.21
Nitrogen	57.67	54.31	9.25
Oxygen	2.95	2.44	1.08
	100.00	100.00	

B

Element	Mass Norm. [%]	Atom [%]	abs. error [%] (1 sigma)
Carbon	33.45	38.45	4.62
Nitrogen	56.49	55.69	8.59
Oxygen	4.74	4.09	1.33
Chlorine	3.18	1.24	0.15
Iron	2.13	0.53	0.10
	100.00	100.00	

C

Element	Mass Norm. [%]	Atom [%]	abs. error [%] (1 sigma)
Carbon	47.16	58.02	1.82
Nitrogen	26.12	27.56	1.33
Oxygen	8.13	7.51	0.48
Chlorine	13.12	5.47	0.16
Iron	5.46	1.45	0.07
	100.00	100.00	

D

Element	Mass Norm. [%]	Atom [%]	abs. error [%] (1 sigma)
Carbon	37.70	42.55	4.90
Nitrogen	55.58	53.80	8.15
Oxygen	2.62	2.22	0.81
Chlorine	3.12	1.19	0.14
Iron	0.97	0.24	0.07
	100.00	100.00	

Figure S6. SEM/EDX analysis of C_3N_4 (A), $[C_3N_4\text{-FeCl(Salen)}]_{Phys}$ (B) and $[C3N4\text{-FeCl(Salen)}]_{Chem}$ (C-D). The $[C_3N_4\text{-FeCl(Salen)}]_{Phys}$ was confirmed to be the physical mixture of the two substances. It is clearly possible to distinguish two primary morphologies assigned respectively to the iron complex (A) and the supporting C_3N_4 (B).

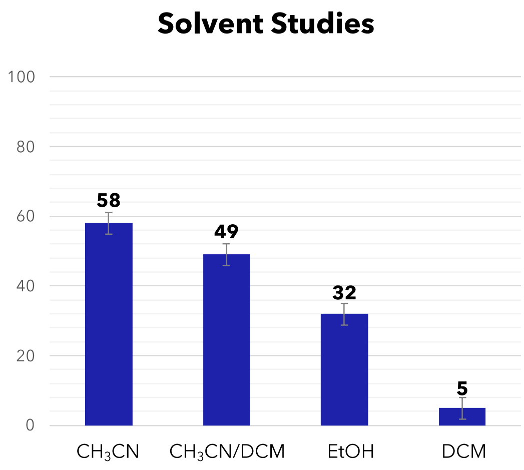

Figure S7. Solvent effect studies on photocatalytic epoxidation of styrene with hydrogen peroxide as oxidant.

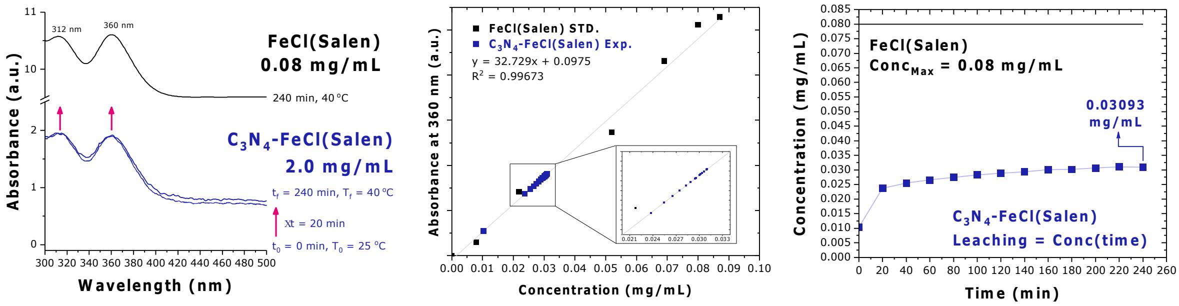

Figure S8. Complex leaching studies with in-situ UV-vis spectroscopy.

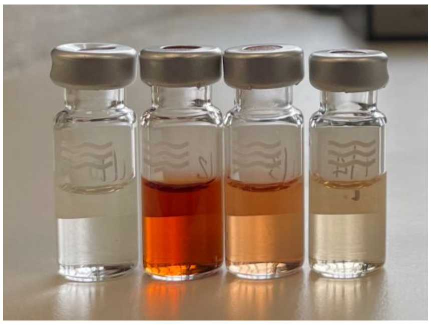

Figure S9. Pictures of styrene oxidation GC test vials after the removal of C_3N_4, $FeSalenCl_2$, $[C_3N_4\text{-FeCl(Salen)}]_{Phys}$ and $[C_3N_4\text{-FeCl(Salen)}]_{Chem}$ respectively from left to right. These correspond to entries 2-5 in Table S5.
